# The Dimensionality of the Brief COPE Before and During the COVID-19 Pandemic

**DOI:** 10.1177/10731911211052483

**Published:** 2021-10-15

**Authors:** Barbara Hanfstingl, Timo Gnambs, Christian Fazekas, Katharina Ingrid Gölly, Franziska Matzer, Matias Tikvić

**Affiliations:** 1 University of Klagenfurt, Klagenfurt am Wörthersee, Austria; 2 Leibniz Institute for Educational Trajectories, Bamberg, Germany; 3 Medical University of Graz, Austria

**Keywords:** Brief COPE, coping, questionnaire, factor structure, higher-order models, COVID-19 pandemic, confirmatory factor analysis

## Abstract

The Brief COPE (Coping Orientation to Problems Experienced) is a frequently used questionnaire assessing 14 theoretically derived coping mechanisms, but psychometric research has suggested inconsistent results concerning its factor structure. The aim of this study was to investigate primary and secondary order factor structures of the Brief COPE during the COVID-19 pandemic by testing 11 different models by confirmatory factor analyses and to assess differences between sex, age groups, and relationship status. Altogether, 529 respondents from Austria and Germany participated in a web-based survey. Results supported the originally hypothesized 14-factor structure but did not support previously described higher-order structures. However, bass-ackwards analyses suggested systematic overlap between different factors, which might have contributed to different factor solutions in previous research. Measurement invariance across sex, age groups, and relationship status could be confirmed. Findings suggest that cultural and situational aspects as well as the functional level should be considered in research on theoretical framing of coping behavior.

Lazarus (1966) defined coping after primary and secondary appraisal as the third of three processes in dealing with stress and threats. These processes are not linear and strictly sequential, as both the threat itself and the functioning of strategies to cope with the threat are appraised and reappraised several times (Carver et al., 1989). As the role of coping behaviors and strategies in overcoming difficulties and resistance is central, interest in developing valid instruments and scales to measure coping is very high, with even greater relevance in times of the COVID-19 pandemic. The aim of this article was to shed light on the structure and dimensionality of one of the most used measurements for coping behavior, the Brief COPE (Coping Orientation to Problems Experienced).

The Brief COPE (Carver, 1997) is a short version of the multidimensional coping inventory (COPE; Carver et al., 1989). The construction rationale of the COPE relied on the works by Lazarus and in parts also on behavioral self-regulation theory by Carver and Scheier (1990). The COPE was theoretically separated into *problem-focused coping* (active coping, planning, suppression of competing activities, restraint coping, seeking of instrumental and social support), *emotion-focused coping* (seeking of emotional and social support, positive reinterpretation, acceptance, denial, turning to religion), and *less useful coping strategies* (focus on and venting of emotions, behavioral disengagement, mental disengagement). Carver (1997) developed the Brief COPE because the original COPE with 14 scales and 53 items (four per scale except for *alcohol-drug disengagement* with only one item) was too long for many applied research projects. The revised instrument was substantially shortened and included some changes in the scales. Some of the original scales that have received little empirical support were omitted; for example, the *suppression of competing activities* scale was empirically redundant with the *active coping* scale (Carver, 1997). Others have been renamed for the Brief COPE; for instance, *mental disengagement* turned to *self-distraction* (see Carver, 1997, for detailed information). Finally, Carver (1997) added a new scale *humor* to the Brief COPE. The final Brief COPE, therefore, consists of 14 scales with two items each (see Appendix A in the Supplemental Material): *acceptance*, *active coping*, *behavioral disengagement*, *denial*, *emotional support*, *humor*, *instrumental support*, *planning*, *positive reframing*, *religion*, *self-blame*, *self-distraction*, *substance use*, and *venting*.

## Brief COPE Dimensionalities

The dimensionality of the Brief COPE has been subject to intensive psychometric research in clinical and non-clinical samples across different cultures (e.g., Bose et al., 2015; Kapsou et al., 2010; Zuckerman & Gagne, 2003). These studies yielded highly inconsistent results regarding a potential higher-order structure for the 14 subscales. For example, Bean and colleagues (2009) published a two-factor solution. The first factor called *approach coping* consisted of the subscales acceptance, active coping, emotional support, instrumental support, planning, positive reframing, and religion. The second factor labeled *avoidance coping* comprised the subscales of behavioral disengagement, denial, humor, self-blame, self-distraction, substance use, and venting. The factor structure published by Mahmoud and colleagues (2012) was consistent with the model by Bean and colleagues (2009), except that the names of the higher-order factors were different (*adaptive* and *maladaptive* instead of *approach* and *avoidance*). In contrast, Paukert and colleagues (2009) identified three higher-order factors, as they modeled a factor with both *substance use* and *religion* scales in addition to *adaptive* and *maladaptive coping*. An adaption of this model was proposed by Eisenberg and colleagues (2012) who excluded the scales of humor and religion as indicators of the higher-order structure. Using data from a German-speaking version, Knoll and colleagues (2005) suggested a four higher-order solution based on the following factors: *positive* (subscales: acceptance, positive reframing, humor), *support* (subscales: emotional support, instrumental support, religion), *evasive coping* (subscales: denial, self-blame, venting), and one higher-order factor consisting of the subscales *active coping* and *planning*. Similarly, Bose and colleagues (2015) proposed a four-factor structure that had structural similarities to the theoretical considerations by Carver and colleagues (1989) and included the factors *problem-focused coping* (subscales: active coping, planning), *avoidant coping* (subscales: substance use, behavioral disengagement, denial), *socially supported coping* (subscales: emotional support, instrumental support, venting), and *emotion-focused coping* (subscales: acceptance, humor, positive reframing, religion). Yet another four-factor solution was proposed by Hastings and colleagues (2005). The factor *active avoidance coping* consisted of one self-distraction item (sd1) and the subscales substance use, venting, and self-blame. The factor *problem-focused coping* comprised the subscales active coping, one emotional support item (es1), instrumental support, and planning. The third higher-order factor *positive coping* contained one acceptance item (ap1), one emotional support item (es2), and the two subscales humor and positive reframing. The two subscales religion and denial represented the last higher-order factor called *religious/denial coping*. Moreover, exploratory factor analyses even lead to a 9-factor solution (Carver, 1997). Here, the subscales substance use, religion, humor, and behavioral disengagement remained as two-item factors. The subscales emotional support and instrumental support represented the higher-order factor *support*. Furthermore, one acceptance item (ap2) and the subscales active coping, planning, and positive reframing build another higher-order factor *active positive coping*. The factor *evasive coping 1* was formed by the subscales venting and self-distraction, whereas the factor *evasive coping 2* consisted of the subscales self-blame and denial. Finally, one acceptance item (ap1) remained as a single-item factor. Table 1 gives an overview of the different higher-order factor structures.

**Table 1.** Higher-Order Loading Structure for Examined Models.

	Scale	Item	Models															
Abbreviations of scales			6	7		8		9			10				11			
			Carver (1997)	Bean et al. (2009)		Eisenberg et al. (2012)		Paukert et al. (2009)			Knoll et al. (2005)				Bose et al. (2015)			
			F1	F1	F2	F1	F2	F1	F2	F3	F1	F2	F3	F4	F1	F2	F3	F4
AP	Acceptance	20.	X	X		X		X			X				X			
		24.	X	X		X		X			X				X			
AC	Active coping	2.	X	X		X		X						X				X
		7.	X	X		X		X						X				X
BD	Behavioral disengagement	6.	X		X		X		X								X	
		16.	X		X		X		X								X	
DE	Denial	3.	X		X		X		X				X				X	
		8.	X		X		X		X				X				X	
ES	Emotional support	5.	X	X		X		X				X				X		
		15.	X	X		X		X				X				X		
HU	Humor	18.	X		X						X				X			
		28.	X		X						X				X			
IS	Instrumental support	10.	X	X		X		X				X				X		
		23.	X	X		X		X				X				X		
PL	Planning	14.	X	X		X		X						X				X
		25.	X	X		X		X						X				X
PR	Positive reframing	12.	X	X		X		X			X				X			
		17.	X	X		X		X			X				X			
RE	Religion	22.	X	X						X		X			X			
		27.	X	X						X		X			X			
SB	Self-blame	13.	X		X		X		X				X					
		26.	X		X		X		X				X					
*SD*	Self-distraction	1.	X		X		X	X										
		19.	X		X		X	X										
SU	Substance use	4.	X		X		X			X							X	
		11.	X		X		X			X							X	
VE	Venting	9.	X		X		X		X				X			X		
		21.	X		X		X		X				X			X		

*Note*. F = higher-order factor; X = scale loaded on higher-order factor. Scales without loading on a higher-order factor were included as first-order factors in the models; **Model 6**: F1 = general coping factor; **Model 7**: F1 = approach coping, F2 = avoidance coping; **Model 8**: F1 = approach coping, F2 = avoidance coping; **Model 9**: F1 = adaptive coping, F2 = maladaptive coping, F3 = religion and substance use; **Model 10**: F1 = positive, F2 = support, F3 = evasive coping, F4 = active coping and planning; **Model 11**: F1 = emotion-focused coping, F2 = socially supported coping, F3 = avoidant coping, F4 = problem-focused coping.

## Brief COPE Factor Structure During the Pandemic

During the COVID-19 pandemic, the original 14-factor structure of Carver (1997) was applied in three studies (Chodkiewicz et al., 2020; Gurvich et al., 2021; Park et al., 2020). Furthermore, several authors also resorted to one of the higher-order models described previously. For example, Rettie and Daniels (2021) used adaptive strategies and maladaptive strategies based on the factors published by Mahmoud and colleagues (2012). In contrast, Fluharty and Fancourt (2021) followed Bose and colleagues (2015) and used the dimensions problem-focused coping, emotion-focused coping, avoidant coping, and socially supported coping, whereas Agha (2021) based her study on the four-factor coping structure by Hastings and colleagues (2005). Importantly, at the moment, there is only one preprint that explicitly examined the dimensionality of the Brief COPE during the pandemic (Rahman et al., 2020). In a sample of 423 female nurses in Brunei, the authors were unable to replicate the model adapted from Carver (1997) and proposed a two-factor solution similar to approach and avoidance coping by Bean and colleagues (2009), but omitting the scale humor.

## Present Study

This study aimed at investigating primary and secondary order factor structures of the Brief COPE during the COVID-19 pandemic. Our goal was to test competing factor models of the Brief COPE with the data collected during the COVID-19 pandemic as part of the Stress and COVID-19 (SC-19) studies^1^ and to detect differences between sex, age groups, and relationship status within the sample. The original instructional text of the Brief COPE focuses on coping behavior over the last 4 weeks. As we were interested in the entire lockdown period from March to May 2020, we adapted it to read, “Please rate the extent to which the following statements were true during the lockdown.”

## Method

### Sample and Procedure

Participants were recruited via snowball sampling by inviting them to participate in an unproctored web-based survey between the beginning of June and mid of August 2020. In Austria and Germany, at that time the participants had experienced the first “lockdown” between mid of March and the beginning of May 2020, with strong restrictions in public and private life. The study examined a convenience sample of *N* = 529 respondents (81% female) from Austria and Germany with complete data sets of the Brief COPE. Their mean age was 36.77 years (*SD* = 13.93). About 75% of them were currently in a relationship (e.g., married), while the remaining respondents indicated to be without a partner. Most participants were highly educated, with about 60% having obtained a university degree. The sociodemographic characteristics of the sample by gender are summarized in Table 2.

**Table 2.** Sample Characteristics.

Abbreviations of scales	Total sample	Women	Men
Sample size	529	423	91
Percentage of women	81%	100%	0%
Mean age (*SD*) in years	36.8 (13.9)	35.9 (13.1)	41.4 (16.9)
Percentage of university degree	60%	61%	60%
Percentage without partner	25%	25%	23%

### Measures

The German version of the Brief COPE (Carver, 1997) administered in this study (Knoll et al., 2005) included 28 items (see Appendix A in the Supplemental Material) measuring 14 theoretically derived coping responses. The respondents were instructed to indicate how strongly the 28 statements applied to their thinking and acting in unpleasant and difficult situations. All responses were given on 4-point response scales using 1 = “not at all,” 2 = “a little bit,” 3 = “a medium amount,” and 4 = “a lot.”

### Statistical Analyses

The dimensionality of the Brief COPE was evaluated using confirmatory factor analyses for ordered categorical variables (e.g., Lei & Shiverdecker, 2020) with a diagonally weighted least square estimator with mean and variance adjusted test statistics. We evaluated 11 different models including various higher-order models that might account for the correlations between the primary factors (see Table 1):

*Model 1*: Carver (1997) hypothesized that the 28 items represented 14 coping responses that were modeled as correlated latent factors with two indicators each.
*Model 2*: Because preliminary analyses indicated a questionable fit of the two items representing active coping, these items were excluded resulting in a model with 26 items representing 13 coping responses.
*Model 3*: In practice, scale scores for the 14 coping responses are typically calculated by averaging the responses to the two items of a scale. However, this implies a specific measurement model. Therefore, constraints were placed on the loadings of the two items for each item in Model 1. A comparable fit as the unconstrained Model 1 would corroborate the use of sum scores as indicators of respondent proficiencies.
*Model 4*: Exploratory factor analyses by Carver (1997) suggested that the self-distracting and venting factors, the use of emotional and instrumental support factors, the denial and self-blame factors, and the active coping, planning, and positive reframing factors might be merged into common factors. Therefore, the model specified nine latent factors, that is, five of the original factors and the four new factors. All factors were allowed to correlate.
*Model 5*: Another exploratory factor analysis (Hastings et al., 2005) suggested a more parsimonious structure including four factors: (a) avoidance coping subsumed the self-distraction, substance use, venting, self-blame, and behavioral disengagement items; (b) religious / denial coping included the positive reframing, religion, and denial items; (c) problem-focused coping referred to the active coping, planning, and use of instrumental support items; and (d) positive coping subsumed the acceptance and humor items. Moreover, the use of emotional support items was split, and one item was included in each of the two latter factors.
*Model 6*: To evaluate whether a general factor might account for all 14 coping responses (Carver, 1997), a second-order factor modeled a single higher-order factor that loaded on all 14 coping factors.
*Model 7*: Bean and colleagues (2009) proposed two higher-order factors: approach and avoidance coping. The former was represented by acceptance, active coping, emotional support, instrumental support, religion, planning, and positive reframing factors. The latter was represented by behavioral disengagement, denial, humor, self-distraction, substance use, self-blame, and venting factors. The two higher-order factors were allowed to correlate. The same factor structure was published by Mahmoud and colleagues (2012), who named the two higher-order factors adaptive and maladaptive coping.
*Model 8*: An adaption of Model 7 was suggested by Eisenberg and colleagues (2012), who excluded the religion and humor factors as indicators of the higher-order factors.
*Model 9*: Paukert and colleagues (2009) identified three correlated higher-order factors. Adaptive coping was represented by the acceptance, active coping, emotional support, instrumental support, planning, positive reframing, and self-distraction factors. Maladaptive coping was modeled using the behavioral disengagement, denial, self-blame, and venting factors. Finally, the religion and substance use factors were modeled by a common higher-order factor.
*Model 10*: Four correlated higher-order factors were suggested by Knoll and colleagues (2005). Focus on the positive was represented by the acceptance, positive reframing, and humor factors, whereas support coping was indicated by the emotional support, instrumental support, and religion factors. Moreover, active coping and planning were modeled using a common higher-order factor. Evasive coping loaded on the denial, self-blame, and venting factors.
*Model 11*: Another four-factor higher-order structure was introduced by Bose and colleagues (2015). They modeled emotion-focused coping using the acceptance, humor, religion, and positive reframing factors. Socially supported coping was represented by the emotional support, instrumental support, and venting factors. Avoidant coping was indicated by the behavioral disengagement, denial, and substance use factors. Again, active coping and planning loaded on a common problem-focused coping factor. All higher-order factors were allowed to correlate.

To further evaluate the hierarchical structure of the coping responses, exploratory bass-ackwards analyses (Goldberg, 2006) were conducted. This involves estimating a series of *varimax* rotated principal component analyses that extracted an increasing number of components and correlating the component scores between different solutions (e.g., between the 10- and 11-component solutions). The input for these analyses was the empirical Bayes estimates of the 14 latent factors in Model 1 (see above). This procedure has gained recent popularity in personality (e.g., Schroeders et al., 2021), intelligence (e.g., Steger et al., 2019), and psychopathological research (e.g., Wright et al., 2012) because it allows exploring the dimensionality of measurement instruments at different levels of abstraction.

Group comparisons require that identical constructs are measured comparably in the different groups (Schroeders & Gnambs, 2020). Therefore, measurement invariance for the 14 coping responses was evaluated following Wu and Eastbrook (2016) on four levels that included increasing constraints. (a) For configural measurement invariance, identical factor models were estimated in all groups (i.e., 14 correlated coping factors) without imposing any constraints across groups. This baseline model evaluated whether the basic factor structure (i.e., number of factors, loading pattern) was comparable in all groups. (b) For weak measurement invariance, the factor loadings were constrained across groups. Because for ordinal factor models with binary or ternary indicators threshold invariance is indistinguishable from configural invariance and, thus, cannot be independently examined (Wu & Eastbrook, 2016), the model also included constraints on all thresholds across groups. Weak invariance is a prerequisite for examining differences in correlations between groups. (c) For strong measurement invariance, additionally the intercepts were constrained across groups, while mean differences in the latent common factors were examined. (d) For strict measurement invariance, thresholds, loadings, intercepts, and specific factor variances were constrained. The latter invariance level allows the comparison of observed scores but is not necessary for latent factor analyses. Model comparisons for the different levels of measurement invariance were based on differences in practical goodness-of-fit indices, namely, the comparative fit index (CFI) and root mean square error of approximation (RMSEA) for which values of ΔCFI ≤.010 and ΔRMSEA ≤.015 are considered negligible differences (Chen, 2007). Because some measurement invariance analyses involved small and imbalanced groups, we replicated these results as a form of sensitivity analyses in a Bayesian framework (Finch et al., 2018; Winter & Depaoli, 2020; Zitzmann et al., 2021). However, these analyses estimated metric factor models because Bayesian multigroup models for ordinal indicators are currently methodologically underdeveloped. To compare the different invariance models, we used the difference in the Bayesian version of the RMSEA (BRMSEA; Garnier-Villarrea & Jorgensen, 2020) and the leave-one-out statistic (LOO; Vehtari et al., 2017). For the former, we considered ΔBRMSEA ≤.015 as negligible differences, whereas LOO differences indicate comparable models if the 95% confidence interval includes 0. The raw data and analysis code generated to produce the reported results are provided at https://osf.io/tkvq8.

## Results

### Response Scale Usage

The items of the Brief COPE were accompanied by 4-point response scales. However, descriptive analyses (see Table 3) showed that the different response options were rather unequally used by the respondents. For example, only 6% of the respondents selected Response Options 3 and 4 of Item 22, whereas Response Options 1 and 2 were chosen by 68% and 20%, respectively. Some items such as Item 13 or 26 exhibited even more pronounced skewed response distributions, resulting in more than 70% or 80% of respondents selecting a given response category. The latter were observed for items of denial, self-blame, and substance use subscales, suggesting rather low prevalence rates of these constructs in the present sample. Because latent variable models with small sample sizes result in rather imprecise parameter estimates, response categories that were selected by less than 10% of the sample were collapsed (Table 3). This resulted in nine items with their original four response categories, 13 items with three response categories, and six dichotomous items.

**Table 3.** Response Scale Usage of the Brief COPE.

Abbreviations of scales	Item	Response categories				Collapsed response categories
		1 (%)	2 (%)	3 (%)	4 (%)	
AP	20	**7**	13	34	46	1,2
	24	**2**	10	36	51	1,2
AC	2	24	38	26	11	
	7	**8**	22	34	36	1,2
BD	6	45	33	15	**7**	3,4
	16	75	13	**9**	**4**	3,4
DE	3	89	**8**	**3**	**1**	2,3,4
	8	78	16	**5**	**2**	2,3,4
ES	5	16	33	30	21	
	15	34	30	19	18	
HU	18	23	32	22	22	
	28	12	40	30	19	
IS	10	41	33	18	**9**	3,4
	23	36	35	20	**9**	3,4
PL	14	11	23	33	33	
	25	10	25	36	29	
PR	12	**4**	17	38	42	1,2
	17	**6**	19	34	42	1,2
RE	22	68	20	**6**	**6**	3,4
	27	62	23	**7**	**8**	3,4
SB	13	77	17	**4**	**3**	2,3,4
	26	84	11	**3**	**2**	2,3,4
*SD*	1	10	19	36	35	
	19	**9**	21	33	36	1,2
SU	4	73	20	**5**	**2**	2,3,4
	11	80	15	**3**	**2**	2,3,4
VE	9	21	37	28	13	
	21	47	32	15	**6**	3,4

*Note*. Reported are the percentages of responses given in each response categories. Response categories selected by less than 10% of the sample (in bold) were collapsed. Abbreviations for scales are given in Table 1. COPE = Coping Orientation to Problems Experienced.

### Evaluation of First-Order Structure

The hypothesized dimensionality of the Brief COPE was evaluated by specifying 14 latent factors represented by two items each that corresponded to the coping responses suggested by Carver (1997). Initial analyses showed a correlation of 1.06 between the active coping and planning factors, suggesting redundant factors; therefore, this correlation was constrained to 1.00. Moreover, the two loadings on the substance use factor were constrained for model identification. Thus, the modified model resulted in a satisfactory fit, χ^2^(261) = 711, CFI = .95, RMSEA = .06, standardized root mean residual (SRMR) = .07. Moreover, all items had substantial loadings on their respective factor (see Model 1 in Table 4).

**Table 4:** Omega Reliabilities (McDonald, 1999) and Factor Loading Pattern for the Brief COPE.

Scale	Item	Omega reliabilities	Model 1	Model 2
AP	20	.67	.69	.78^a^
	24		.89	.78^a^
AC	2	.42	.51	.58^a^
	7		.63	.58^a^
BD	6	.37	.48	.57^a^
	16		.67	.57^a^
DE	3	.73	.78	.87^a^
	8		.97	.87^a^
ES	5	.74	.75	.80^a^
	15		.86	.80^a^
HU	18	.71	.74	.80^a^
	28		.86	.80^a^
IS	10	.84	.87	.91^a^
	23		.95	.91^a^
PL	14	.51	.74	.62^a^
	25		.51	.62^a^
PR	12	.77	.85	.86^a^
	17		.88	.86^a^
RE	22	.77	.86	.87^a^
	27		.89	.87^a^
SB	13	.61	.88	.82^a^
	26		.75	.82^a^
*SD*	1	.58	.52	.68^a^
	19		.88	.68^a^
SU	4	.90	.98^a^	.98^a^
	11		.98^a^	.98^a^
VE	9	.46	.29	.29
	21		.92	.92

*Note*. Reported are standardized factor loadings for two models. Abbreviations for scales are given in Table 1. COPE = Coping Orientation to Problems Experienced.

a Constrained loadings for factor.

These results support the originally hypothesized structure to a large degree, albeit pointing to a lack of discriminant validity between the active coping and planning factors (see Table 5 for the respective correlations). Modification indices suggested substantial cross-loadings of the two items representing active coping on denial and self-blame. Given the multicollinearity of the active coping factor with planning (see above), another model was evaluated that excluded the active coping items. The model with only 13 coping strategies resulted in a slightly better fit, χ^2^(222) = 541, CFI = .96, RMSEA = .05, SRMR = .06, giving further support for 13 distinct coping factors in the Brief COPE. Finally, a third model was evaluated that constrained the two factor loadings for each coping factor.^2^ This model evaluated whether the prevalent practice of simply averaging the item responses to create scale scores seems justified (McNeish & Wolf, 2020).

**Table 5.** Latent Correlations of the Coping Responses of the Brief COPE.

Abbreviations of scales	AP	AC	BD	DE	ES	HU	IS	PL	PR	RE	SB	*SD*	SU	VE
AP	—													
AC	.16*	—												
BD	−.08	−.43*	—											
DE	−.53*	.00	.42*	—										
ES	.03	.39*	−.18*	.14	—									
HU	.38*	.23*	.09	−.07	.17*	—								
IS	−.07	.34*	−.08	.25*	.82*	−.03	—							
PL	.26*	1.00^a^	−.33*	.01	.48*	.16*	.51*	—						
PR	.59*	.57*	−.21*	−.27*	.15*	.47*	.04	.43*	—					
RE	−.16*	.43*	.03	.28*	.21*	.02	.29*	.34*	.29*	—				
SB	−.27*	−.13	.46*	.40*	.13	−.04	.33*	.08	−.29*	.13	—			
SD	.25*	.52*	−.19*	.11	.38*	.34*	.21*	.44*	.24*	.06	−.02	—		
SU	−.15*	−.16	.24*	.28*	.24*	−.03	.18*	−.10	−.22*	−.07	.27*	.09	—	
VE	−.16*	.18*	.26*	.33*	.62*	.11	.63*	.23*	−.12	.20*	.30*	.23*	.27*	—

*Note*. Abbreviations for scales are given in Table 1. COPE = Coping Orientation to Problems Experienced.

a Constrained parameter.

* *p* < . 05.

The constrained model (Model 3 in Table 6) resulted in a negligible deterioration of fit compared with the unconstrained model. Thus, the assumption of equal factor loading for both items of each factor was corroborated. The omega reliabilities (McDonald, 1999) of the 14 factors are summarized in Table 4. Despite their short length with only two items, most factors exhibited good reliabilities between .70 and .90. In contrast, three scales (active coping, behavioral disengagement, venting) showed rather low reliabilities between .37 and .46. However, it has been argued that conventional thresholds of reliability should not be used for ultra-short scales because these prioritize construct breadth over measurement precision (Rammstedt & Beierlein, 2014).

**Table 6:** Goodness of Fit for Confirmatory Factor Models of the Brief COPE.

Model	χ^2^(*df*)	CFI	RMSEA	SRMR	Model comparisons			Reliability of higher-order factors
					ΔCFI	ΔRMSEA	ΔSRMR	
1. Unconstrained 14 primary factors (Carver, 1997)	711 (261)	.95	.06	.07				
2. Unconstrained 13 primary factors (excluding “active coping”)	541 (222)	.96	.05	.06				
3. Constrained 14 primary factors	782 (273)	.94	.06	.07	.01	.00	.00	
4. Nine primary factors (Carver, 1997)	1,322 (318)	.89	.08	.10	.06	.02	.03	
5. Four primary factors (Hastings et al., 2005)	2,915 (344)	.71	.12	.17	.14	.06	.10	
6. Single second-order factor	1,811 (349)	.84	.09	.14	.11	.03	.07	.67
7. Two second-order factors (Bean et al., 2009)	1,812 (348)	.84	.09	.14	.11	.03	.07	.70/.33
8. Two second-order factors (Eisenberg et al., 2012)	1,832 (345)	.84	.09	.14	.11	03	.07	.70/.30
9. Three second-order factors (Paukert et al., 2009)	1,980 (347)	.82	.09	.15	.13	.03	.08	.70/.69/.38
10. Four second-order factors (Knoll et al., 2005)	1,005 (334)	.93	.06	.10	.02	.00	.03	.72/.69/.68/.51
11. Four second-order factors (Bose et al., 2015)	1,114 (338)	.91	.07	.11	.04	.01	.04	.81/.69/.61/.48

*Note*. All model comparisons use Model 1 as the reference model. COPE = Coping Orientation to Problems Experienced; CFI = comparative fit index; RMSEA = root mean square error of approximation; SRMR = standardized root mean residual.

### Evaluation of Second-Order Structure

Some factors were substantially correlated (see Table 5). For example, acceptance and positive reframing correlated at .59, whereas emotional and instrumental support correlated at .82. Therefore, previous research suggested different higher-order structures of the Brief COPE that might account for these inter-correlations. The respective fits for six different second-order models are summarized in Table 6. These results show that all higher-order models had a worse fit than the correlated 14 (and 13) factor models; in most instances, the deterioration of model fit was substantial (i.e., ΔCFIs around .11 and ΔRMSEAs around .03). The best fit was achieved by the second-order model proposed by Knoll and colleagues (2005) that modeled the four higher-order factors *focus on the positive* (represented by the acceptance, positive reframing, and humor factors), *support coping* (represented by the use of emotional support, use of instrumental support, and religion factors), *active coping* (represented by the active coping and planning factors), and *evasive coping* (represented by the self-blame, denial, and venting factors). However, the reliability of the latter was somewhat limited (ω = .51). Overall, these results gave limited support for a systematic higher-order structure of the Brief COPE.

### Exploratory Hierarchical Analyses

**Figure 1:**
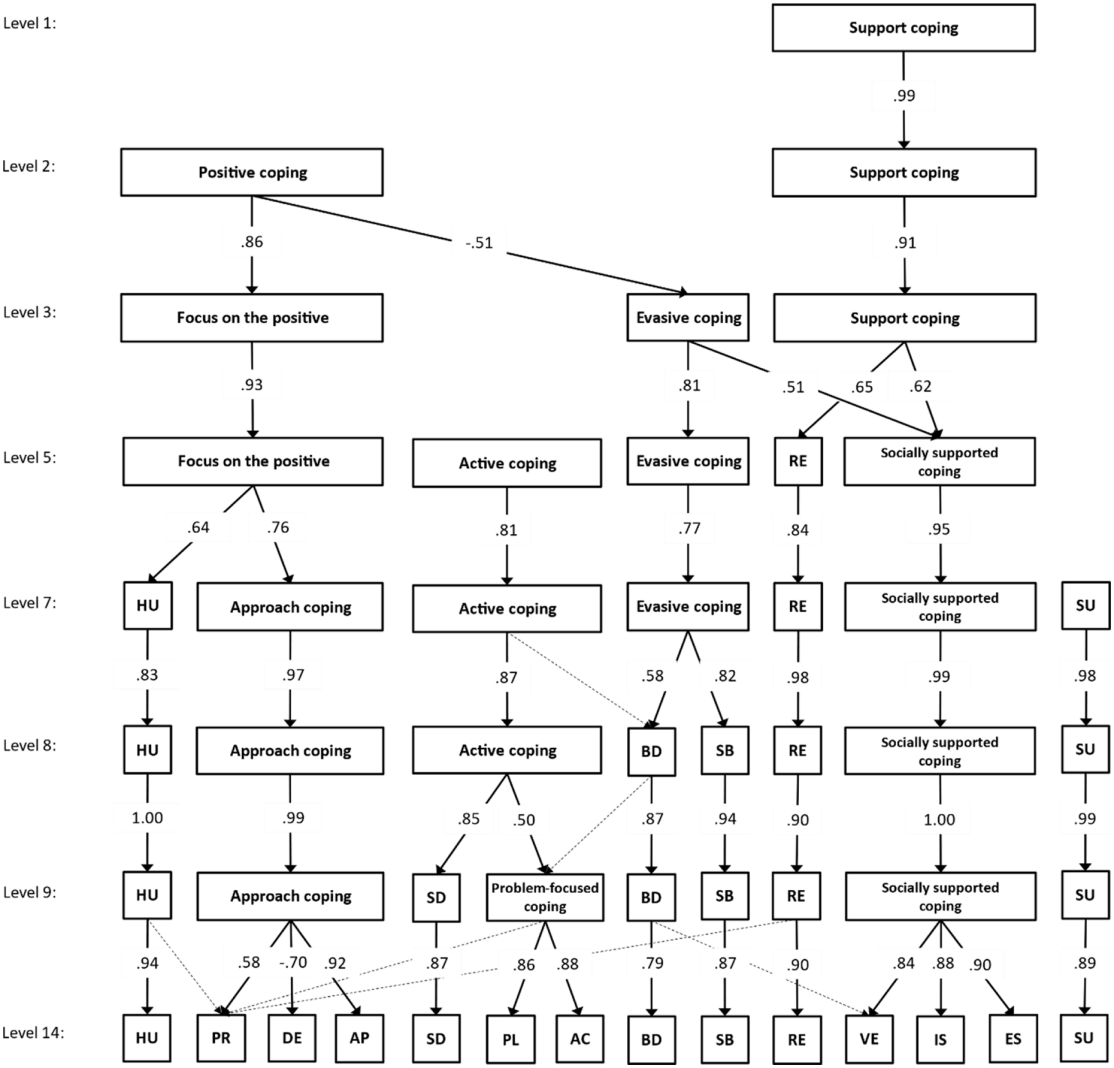
Correlations Between Factor Scores on the Different Levels of the Dimensional Hierarchy of Coping Responses *Note.* Correlations below .40 were omitted and correlations between .40 and .50 are displayed as dashed lines. Omitted levels exhibited no new higher-order factors with correlations greater than .50. Abbreviations of primary factors are given in Table 1.

Exploratory analyses tried to determine a hierarchical structure of coping responses on different levels of abstraction (see Goldberg, 2006). To this end, several orthogonally rotated principal component analyses were conducted. The correlations between factor scores from different hierarchical levels are presented in Figure 1. Correlations below .40 are not presented. Moreover, hierarchical levels including principal components without loadings exceeding .50 were excluded. As shown in Figure 1, the first higher-order structure was represented by nine components, six of which were identical to the primary coping responses. Similar to previous research (Bose et al., 2015; Knoll et al., 2005), one component merged the active coping and planning factors (i.e., problem-focused coping). Another component was represented by the use of emotional support, use of instrumental support, and venting factors, which was identical to the second-order factor “socially supported coping” suggested by Bose and colleagues (2015). Finally, a third component combined the approach, denial, and positive reframing factors. Although this higher-order structure has not been previously identified, the two former factors frequently loaded on a common second-order factor in previous research (Bose et al., 2015; Knoll et al., 2005). Together these results suggest similar higher-order components as identified in previous research. On the eighth level, problem-focused coping was combined with self-distraction, whereas the seventh level combined behavioral disengagement and self-blame. However, in both cases, the higher-order component was dominated by one factor, whereas the other factor showed a smaller correlation. Further dimensional reductions below level 7 were generally unsatisfactory because they either did not represent all 14 primary factors anymore or did not exhibit a simple structure, making the interpretation of the higher-order components difficult.

Taken together, these results suggest that the 14 coping responses might be summarized by seven to nine higher-order components. The best differentiation was achieved for the approach coping, problem-focused coping, and social support coping higher-order components. However, it needs to be emphasized that the positive reframing factor exhibited substantial cross-correlations on different higher-order components (e.g., with problem-focused coping, religion, humor). Thus, it might be speculated that this coping strategy represents a rather central component that, to some degree, is also important for various other coping strategies.

### Measurement Invariance

Measurement invariance for the 14 coping responses was evaluated for three criteria: (a) sex (women vs. men), (b) age groups (young = 18–34 years vs. middle / old = 35 years and older), and (c) relationship status (with vs. without a partner). The four levels of configural, weak, strong, and strict measurement invariance were independently estimated for each criterion. The respective model fits for these analyses are summarized in Table 7. These results show that for all three criteria, even strict measurement invariance can be assumed because none of the model comparisons revealed a loss of fit exceeding ΔCFI ≤.010, ΔRMSEA ≤.015, or ΔBRMSEA ≤.015. Also, the Bayesian LOO statistic corroborated strong measurement invariance for the three criteria. Thus, the Brief COPE exhibited measurement invariance across sex, age groups, and relationship status, which allows mean-level comparisons across groups. The respective mean differences (as Cohen’s *d*s) for each of the 14 coping responses are given in Table 8. These results showed that men had significantly (*p* < .05) higher coping responses on humor (*d* = 0.61) and substance use (*d* = 0.98), whereas women exhibited higher responses on instrumental support (*d* = −0.38), positive reframing (*d* = −0.28), and religion (*d* = −0.47). Similarly, younger respondents adopted the strategies of behavioral disengagement (*d* = 0.65), emotional support (*d* = 0.43), instrumental support (*d* = 0.38), self-blame (*d* = 0.91), self-distraction (*d* = 0.37), and venting (*d* = 0.57) to a larger degree, whereas older respondents had more pronounced coping responses on active coping (*d* = −0.32) and positive reframing (*d* = −0.26). In contrast, differences between singles and respondents with a partner were limited to behavioral disengagement (*d* = 0.90) and self-blame (*d* = 0.63). For the latter, however, it is unclear to what degree differences between relationship status are confounded with age differences.

**Table 7.** Measurement Invariance of the Brief COPE Across Sex, Age Groups, and Relationship Status.

Model	Frequentist analyses					Bayesian analyses			
	χ^2^(*df*)	CFI	RMSEA	Model comparisons		BRMSEA	LOO	Model comparisons	
				ΔCFI	ΔRMSEA			ΔBRMSEA	ΔLOO (95% CI)
Sex: men (*N* = 91) versus women (*N* = 423)									
1. Configural invariance	941 (548)	.953	.053			.053	32,611		
2. Weak invariance	951 (556)	.952	.053	.001	.000	.054	32,607	.001	−2.605 [−14.438, 9.228]
3. Strong invariance	989 (572)	.950	.053	.002	.000	.056	32,642	.003	−15.276 [−35.123, 4.571]
4. Strict invariance	1,012 (591)	.949	.053	.004	.000	.057	32,637	.004	−12.386 [−46.837, 22.065]
Age groups: young (*N* = 279) versus middle / old adulthood (*N* = 237)									
1. Configural invariance	988 (548)	.952	.056			.051	32650		
2. Weak invariance	978 (556)	.954	.054	.000	.000	.050	32,631	−.001	−9.145 [−20.793, 2.503]
3. Strong invariance	1,023 (570)	.951	.056	.001	.000	.053	32,665	.002	−7.577 [26.564, 11.410]
4. Strict invariance	1,081 (591)	.947	.057	.005	.000	.060	32,763	.009	−56.291 [−109.291, −3.433]
Relationship status: with (*N* = 370) versus without partner (*N* =155)									
1. Configural invariance	986 (548)	.947	.055			.052	33,422		
2. Weak invariance	1,010 (556)	.945	.056	.002	.000	.052	33,416	.000	−3.161 [−15.634, 9.312]
3. Strong invariance	1,031 (570)	.944	.056	.003	.000	.053	33,421	.001	−0.834 [−17.719, 16.051]
4. Strict invariance	1,043 (591)	.945	.054	.002	.001	.054	33,412	.002	−5.005 [−35.879, 24.869]

*Note*. All model comparisons use Model 1 as the reference model. COPE = Coping Orientation to Problems Experienced; CFI = comparative fit index; RMSEA = root mean square error of approximation; BRMSEA = Bayesian root mean square error of approximation (Garnier-Villarrea & Jorgensen, 2020); LOO = leave-one-out statistic (Vehtari et al., 2017); CI = confidence interval.

**Table 8:** Latent Mean Group Differences in Coping Responses.

Abbreviations of scales	Sex (men vs. women)	Age group (young vs. middle / old)	Partner (no vs. yes)
AP	0.25	0.08	−0.05
AC	−0.22	−0.32*	−0.11
BD	−0.18	0.65*	0.90*
DE	−0.32	0.01	−0.12
ES	−0.20	0.43*	0.05
HU	0.61*	0.02	0.18
IS	−0.38*	0.38*	0.04
PL	−0.11	−0.11	−0.11
PR	−0.28*	−0.26*	−0.19
RE	−0.47*	−0.26	−0.09
SB	−0.43	0.91*	0.63*
*SD*	−0.19	0.37*	0.08
SU	0.98*	0.35	−0.17
VE	−0.32	0.57*	−0.02

*Note*. Reported are standardized mean differences *d*. Positive values indicate a larger mean in the first group, whereas negative values indicate a larger mean in the second group. Abbreviations for scales are given in Table 1.

* *p* < .05.

## Discussion

The reported analyses of the German version of the Brief COPE (Knoll et al., 2005) do not only confirm measurement invariance across sex, age groups, and relationship status but also Carver’s (1997) theoretically derived original 14-factor solution. At the same time, no higher-order factor structure previously described in the literature could be substantiated. It seems that the Brief COPE is best represented by its original form without combining the subscales into higher-order units. However, detailed results confirm Carver’s (1997) initial findings, for example, that several scales exhibit substantial multicollinearity. In our data, we found high redundancies of the scales active coping and planning, which also led to a better fit when excluding the active coping items from the analysis. Although we were unable to corroborate any a priori hypothesized higher-order structure, an exploratory evaluation of the hierarchical structure in terms of bass-ackwards analyses (Goldberg, 2006) reflected similar results in previous studies (Figure 1; for example, problem-focused coping like in Models 4 and 11; socially supported coping like in Model 11). Thus, the previously described higher-order structures of the Brief COPE seem to be sample-specific and are not robust across different respondent groups. To some extent, the higher-order structure might also be linguistically and culturally predetermined, as the best fit for a higher-order model was observed for a model that was previously identified in a German-language version of the Brief COPE (Knoll et al., 2005). Knoll and her colleagues used their German-language version in a study focusing on coping with cataract surgeries, hence in a completely different context and time as we used it. Although we were closer to contexts and time of other studies during the pandemic (Model 5 by Hastings et al., 2005; Model 7 by Bean et al., 2009; Model 11 by Bose et al., 2015), no other hierarchical model had a better fit than the model by Knoll and colleagues (2005). In future studies, more space should be given to a systematic investigation of language and culture specificity of higher-order structures of coping behavior measured by the Brief COPE.

Another problem with the Brief COPE is a conceptual confusion because no consistent terminology is used for higher-order factors of the Brief COPE. Sometimes, higher-order factors with the same name were composed of different primary factors (e.g., Bean et al., 2009; Eisenberg et al., 2012), and sometimes factors were named differently despite including the same primary factors (e.g., Bean et al., 2009; Mahmoud et al., 2012). This indicates a jingle-jangle fallacy. A jingle fallacy is the erroneous assumption that two or more measures with the same name cover the same construct, whereas a jangle fallacy represents the misconception that two or more measures with different names automatically refer to two different constructs. Kelley (1927) was the first to describe this phenomenon when he discussed the measurement of achievement and intelligence. Hagger (2014) point to a very similar problem for several variables in social psychology (e.g., self-control) and called it *déjà-variable phenomenon.* Epistemological considerations on this topic and the consequences for research have been discussed by Hanfstingl (2019). Coping research lacks theoretical considerations, systematic investigation, and operationalization across cultures, languages, and even situational and contextual dependences of coping behaviors. This problem gets obvious with the scales of humor and religion: Sometimes, they refer to adaptive factors, sometimes to maladaptive factors, and sometimes to independent factors. In our exploratory hierarchical analyses, these two factors stand a long time alone without merging with other factors (Figure 1). The Brief COPE items do not distinguish between positive and negative religious coping styles or between adaptive humor and maladaptive humor, which does make a difference for health outcomes for both religious coping (e.g., Francis et al., 2019; Park et al., 2018) and humor (e.g., Dozois et al., 2009; Panichelli et al., 2018). We assume that this undetected jingle-jangle fallacy led to an almost arbitrary selection of different factor structures and hierarchies as well as sample-dependent selective omission of single factors (see also Bose et al., 2015, Table 3). For example, in our study, Model 2 with 13 factors, in which active coping was omitted due to multicollinearity with planning, shows a better fit than the 14-factor solution (Model 1). From a theoretical perspective, however, we would not argue that active coping and planning represent the same coping behavior. In our opinion, the problem lies at the operational level.

Probably, the exploratory hierarchical analyses in Figure 1 could be a first step to systematize higher-order solutions of the Brief COPE. A further promising starting point could be theoretical considerations when developing the COPE (Carver et al., 1989) with the higher-order factors problem-focused coping, emotion-focused coping, and less useful coping strategies. According to this, one helpful theory could be the action versus state orientation approach (e.g., Groß, 2020; Schlinkert & Koole, 2018) because it provides a well-integrated theoretical structure for coping behavior in general.

Some of the findings may have their origins in the COVID-19 pandemic. For example, due to the uneven use of response options on the 4-point Likert-type scale, we decided to merge those options that were used by less than 10% of the respondents. This did not affect all scales of the Brief COPE to the same extent. The scales most affected were denial, self-blame, and substance use, that is, those that represent maladaptive coping strategies in particular. In addition to basic social desirability biases, this may also be caused by the need to trivialize the problem; the actual extent of the disaster in a psychological sense was not yet clear in early 2020. A first study in China indicates a delay in the psychological consequences of the pandemic (Gan et al., 2020). Future studies will shed light on this question.

Further results seem to be typical for the pandemic. We identified age differences in the scales active coping and positive reframing, with higher levels reported by participants with older age, while younger participants reported higher values in behavioral disengagement, emotional support, self-blame, self-distraction, and venting. These results go in line with findings in several studies. Although at the beginning of the pandemic older people were expected to feel more threatened and stressed due to their higher risk status (Applegate & Ouslander, 2020; Jordan et al., 2020), several studies proved the opposite. They confirm that younger people have more problems with the COVID-19 pandemic due to feelings of higher stress and uncertainty (Birditt et al., 2021; Bruine de Bruin et al., 2021; Gerhold, 2020; Park et al., 2020). Other studies identified certain factors such as young age, female sex, socioeconomic status, or having underage children that led to particular burden (e.g., Liang et al., 2020; Orgilés et al., 2020; Park et al., 2020; Pearman et al., 2021). Moreover, we observed significant differences in the behavioral disengagement and self-blame scales with respect to relationship status (singles report higher scores). Males reported higher values in the scales humor and substance use, whereas females showed higher scores on instrumental support, positive reframing, and religion, which goes in line with the findings by Park and colleagues (2020) in the United States, Agha (2021) in Saudi Arabia, Chodkiewicz and colleagues (2020) in Poland, Gurvich and colleagues (2021) in Australia, and Rettie and Daniels (2021) in the United Kingdom.

### Limitations

Although the analyses of the Brief COPE have led to important insights into the shortcomings of the theoretical foundation of coping behavior, several limitations still need to be mentioned here. Because the sample was created through a snowball system, it cannot be assumed to be representative. This can also be seen in the overrepresentation of women and people with higher education. Therefore, the results of our analyses cannot be generalized to more heterogeneous populations. However, as noted in the “Discussion” section, there is a lack of clear theoretical foundations and systematizations in the study of coping behavior. This goal should be achieved before further empirical analyses.

## Conclusion

This study has shown that the primary order structure of the Brief COPE with 14 scales works well and even shows strict measurement invariance for sex, age, and relationship status, while no higher-order structure could be verified. We ascribe this to a high dependence on linguistic and cultural influences, jingle-jangle fallacies, and concept vagueness. These findings also substantiate the wide spectrum of somewhat discrete behavioral coping options. Taken together, these findings indicate that, despite the theoretical underpinnings of Carver and colleagues (1989), there is a lack of broader theoretical framing of what coping behavior exactly means and how it relates to individual, cultural, and situational aspects. There is, therefore, a definite need for a theoretical framing of research on coping behavior. Psychological research offers helpful approaches, such as the distinction between problem-oriented and emotion-oriented coping, or the study of action and state orientation, which aim to look at a functional rather than just descriptive level.

## Supplemental Material

sj-docx-1-asm-10.1177_10731911211052483 – Supplemental material for The Dimensionality of the Brief COPE Before and During the COVID-19 Pandemic Click here for additional data file. Supplemental material, sj-docx-1-asm-10.1177_10731911211052483 for The Dimensionality of the Brief COPE Before and During the COVID-19 Pandemic by Barbara Hanfstingl, Timo Gnambs, Christian Fazekas, Katharina Ingrid Gölly, Franziska Matzer and Matias Tikvić in Assessment

## Notes

1. The Stress and COVID-19 (SC-19) studies were started in cooperation with the Medical University Graz and the University of Klagenfurt during the first hard lockdown in the European German-speaking countries. The aim of the studies is to investigate resilience, psychosomatic competence, stress load, and their influence on coping behavior and health during and after the COVID-19 lockdown. In this substudy, the following instruments were applied in addition to the Brief COPE: A German version of the *Fear of Covid Scale* (Ahorsu et al., 2020), German short version of the *Connor-Davidson Resilience Scale* (Sarubin et al., 2015) to measure trait resilience, the German version of the *Patient Health Questionnaire* (Gräfe et al., 2004) to measure depressiveness, the German version of the *Generalized Anxiety Disorder Screener* (Hinz et al., 2017) to measure anxiety, the *Psychosomatic Competence Inventory* (Fazekas et al., 2020), and the *Somatic Symptom Scale–8* (Gierk et al., 2014). This article focuses only on the analyses of the Brief COPE.

2. No constraints were placed on the loadings of the venting factor because preliminary analyses did not support respective constraints.
