# Supplementary material for: The Dimensionality of the Brief COPE Before and During the COVID-19 Pandemic
Source: Assessment. 2021 Oct 15;30(2):287–301. doi: 10.1177/10731911211052483 (PMC9902999; doi:10.1177/10731911211052483)
Supplement: sj-docx-1-asm-10.1177_10731911211052483 – Supplemental material for The Dimensionality of the Brief COPE Before and During the COVID-19 Pandemic [file sj-docx-1-asm-10.1177_10731911211052483.docx]

**APPENDIX A**

*The 28-item Brief COPE Scale by Carver (1997) in English and German*

|  | English | German | Scale |
| --- | --- | --- | --- |
| 1. | I’ve been turning to work or other activities to take my mind off things. | Ich habe mich mit Arbeit oder anderen Sachen beschäftigt, um auf andere Gedanken zu kommen. | Self-distraction |
| 2. | I’ve been concentrating my efforts on doing something about the situation I'm in. | Ich habe mich darauf konzentriert, etwas an meiner Situation zu verändern. | Active coping |
| 3. | I’ve been saying to myself "this isn't real.". | Ich habe mir eingeredet, dass das alles nicht wahr ist. | Denial |
| 4. | I’ve been using alcohol or other drugs to make myself feel better. | Ich habe Alkohol oder andere Mittel zu mir genommen, um mich besser zu fühlen. | Substance use |
| 5. | I’ve been getting emotional support from others. | Ich habe aufmunternde Unterstützung von anderen erhalten. | Use of emotional support |
| 6. | I’ve been giving up trying to deal with it. | Ich habe es aufgegeben, mich damit zu beschäftigen. | Behavioral disengagement, |
| 7. | I’ve been taking action to try to make the situation better. | Ich habe aktiv gehandelt, um die Situation zu verbessern. | Active coping |
| 8. | I’ve been refusing to believe that it has happened. | Ich wollte einfach nicht glauben, dass mir das passiert. | Denial |
| 9. | I’ve been saying things to let my unpleasant feelings escape. | Ich habe meinen Gefühlen freien Lauf gelassen. | Venting |
| 10. | I’ve been getting help and advice from other people. | Ich habe andere Menschen um Hilfe und Rat gebeten. | Use of instrumental support |
| 11. | I’ve been using alcohol or other drugs to help me get through it. | Um das durchzustehen, habe ich mich mit Alkohol oder anderen Mitteln besänftigt. | Substance use |
| 12. | I’ve been trying to see it in a different light, to make it seem more positive. | Ich habe versucht, die Dinge von einer positiveren Seite zu betrachten. | Positive reframing |
| 13. | I’ve been criticizing myself. | Ich habe mich selbst kritisiert und mir Vorwürfe gemacht. | Self-blame |
| 14. | I’ve been trying to come up with a strategy about what to do. | Ich habe versucht, mir einen Plan zu überlegen, was ich tun kann. | Planning |
| 15. | I’ve been getting comfort and understanding from someone. | Jemand hat mich getröstet und mir Verständnis entgegengebracht. | Use of emotional suppor |
| 16. | I’ve been giving up the attempt to cope. | Ich habe gar nicht mehr versucht, die Situation in den Griff zu kriegen. | Behavioral disengagement, |
| 17. | I’ve been looking for something good in what is happening. | Ich habe versucht, etwas Gutes in dem zu finden, was mir passiert ist. | Positive reframing |
| 18. | I’ve been making jokes about it. | Ich habe Witze darüber gemacht. | Humor |
| 19. | I’ve been doing something to think about it less, such as going to movies, watching TV, reading, daydreaming, sleeping, or shopping. | Ich habe etwas unternommen, um mich abzulenken. | Self-distraction |
| 20. | I’ve been accepting the reality of the fact that it has happened. | Ich habe mich damit abgefunden, dass es passiert ist. | Acceptance |
| 21. | I’ve been expressing my negative feelings. | Ich habe offen gezeigt, wie schlecht ich mich fühle. | Venting |
| 22. | I’ve been trying to find comfort in my religion or spiritual beliefs. | Ich habe versucht, Halt in meinem Glauben zu finden. | Religion |
| 23. | I’ve been trying to get advice or help from other people about what to do. | Ich habe versucht, von anderen Menschen Rat oder Hilfe einzuholen. | Use of instrumental support |
| 24. | I’ve been learning to live with it. | Ich habe gelernt, damit zu leben. | Acceptance |
| 25. | I’ve been thinking hard about what steps to take. | Ich habe mir viele Gedanken darüber gemacht, was hier das Richtige wäre. | Planning |
| 26. | I’ve been blaming myself for things that happened. | Ich habe mir für die Dinge, die mir widerfahren sind, selbst die Schuld gegeben. | Self-blame |
| 27. | I’ve been praying or meditating. | Ich habe gebetet oder meditiert. | Religion |
| 28. | I’ve been making fun of the situation. | Ich habe alles mit Humor genommen. | Humor |
| *Note*. The items of the English (Carver, 1997) and German (Knoll et al., 2005) version of the Brief COPE are presented with four-point response scales: 1 = “I haven’t been doing this at all” / “überhaupt nicht”, 2 = “I’ve been doing this a little bit” / “ein bisschen”, 3 = “I’ve been doing this a medium amount” / “ziemlich”, 4 = “I’ve been doing this a lot” / “sehr”. | | | |
